# Corticophobia among Parents of Children with Atopic Dermatitis: Assessing Major and Minor Risk Factors for High TOPICOP Scores

**DOI:** 10.3390/jcm12216813

**Published:** 2023-10-27

**Authors:** Astrid Herzum, Corrado Occella, Lodovica Gariazzo, Carlotta Pastorino, Gianmaria Viglizzo

**Affiliations:** Dermatology Unit, U.O.C. Dermatologia e Centro Angiomi, IRCCS Istituto Giannina Gaslini, Via Gerolamo Gaslini 5, 16147 Genova, Italy; corradooccella@gaslini.org (C.O.); lodovicagariazzocelesia@gaslini.org (L.G.); carlottapastorino@gaslini.org (C.P.); gianmariaviglizzo@gaslini.org (G.V.)

**Keywords:** corticophobia, topical corticosteroids, eczema, atopic dermatitis, topical treatment, pediatric, parental education, parental corticophobia

## Abstract

Corticophobia, fear of applying topical corticosteroids (TCSs), is a rising issue in industrialized countries, despite the actual safety of TCSs for atopic dermatitis (AD). Patients attending the Pediatric Dermatology Unit for skin examination were screened for AD. AD patients were included, and data were collected. Parental corticophobia was evaluated through the Topical Corticosteroid Phobia (TOPICOP) questionnaire. The χ^2^ test and logistic regression were used to analyze statistical associations between parental corticophobia (mild/moderate vs. severe) and patients’ and parents’ characteristics. Overall, 100 patients were included (53 females; 47 males; mean age 5.9 years): 44 had mild/moderate AD (EASI ≤ 21), and 56 had severe AD (EASI > 21) (mean EASI 19.7). Of the patients, 33 never consulted healthcare providers for AD, and 67 did. Parental education was low/intermediate in 60 cases and high (gymnasium/university degree) in 40. Mean parental DLQI was 10.7. Mean parental TOPICOP was 39.1%: 51 had mild/moderate corticophobia (TOPICOP ≤ 50%), and 49 had severe corticophobia (TOPICOP > 50%). At the χ^2^ test, corticophobia was associated with mild/moderate AD (OR 20.9487; 95% CI 7.2489–60.5402; *p* < 0.001), older age of patients (OR 4.1176; 95% CI 1.7880 to 9.4828; *p* < 0.001), early disease onset (OR 9.8925; 95% CI 2.7064–36.1596; *p* < 0.001), and previous healthcare professional consultations (OR 4.9279; 95% CI 1.9335–12.5597; *p* < 0.001). Also, severe parental corticophobia was very significantly associated with severe parental involvement of life quality (OR 33.3333; 95% CI 10.9046–101.8937; *p* < 0.001) and with high education of parents (gymnasium or university degree) (29/49) (OR 5.2727; 95% CI 2.1927–12.6790; *p* < 0.001). At logistic regression, high parental DLQI (*p* < 0.0001), high parental education (*p* < 0.0338), older age of patients (*p* = 0.0015), and early disease onset (*p* < 0.0513) accounted for major risk factors influencing severe parental corticophobia. Assessing risk factors for corticophobia is essential for addressing groups of parents at higher risk for corticophobia using educational programs, to overcome unfounded fears and augment treatment adherence.

## 1. Introduction

Atopic dermatitis (AD) is a chronic inflammatory disease, with highest prevalence among children, requiring daily treatment with emollients. AD is characterized by periodic flare-ups that require also application of topical corticosteroids (TCSs) to control inflammation [1,2]. However, low adherence rates to TCSs are frequently observed, mostly as a consequence of concerns about possible side effects deriving from TCSs. Indeed, patients and their parents are often worried about overusing TCSs and about possible rebound effects arising when stopping their use [1]. Overall, this leads to a generalized fear of applying a TCS, called corticophobia. The phobia of TCSs is generally promoted by misinformation and involves erroneous beliefs and vague negative feelings about TCSs, held by patients and their parents. It represents a common concern among patients with AD and their parents, despite the actual safety and effectiveness of TCS treatment of AD [3]. Indeed, a rate of 21.0–83.7% of TCS phobia was reported among AD patients in a recent systematic review of the literature [4]. As expected, in two studies comparing therapeutical nonadherence between corticophobics and non-corticophobics, patients in the corticophobic group showed significantly higher nonadherence rates to topical therapy with TCS than non-corticophobics (49.4% vs. 14.1% and 29.3% vs. 9.8%) [5,6]. Possible causative factors for corticophobia include misinformation and conflicting advice from healthcare providers, higher number of general practitioner visits, polypharmacy, frequent changing of dermatology clinics, higher income, higher education, urban residency, lack of education about TCSs, fear of adverse effects of TCSs, and personally experienced side effects [7,8,9]. Yet, it seems that the actual severity of disease does not particularly influence the severity of corticophobia; it is rather influenced by the personal perception of disease [8]. Also, it must be considered that a high number of messages about the ‘risk’ derived from TCSs is delivered daily from family, friends, the internet, TV, and other broadcasting media [9,10]. These possibly affect the understanding about the safety of TCSs and may ultimately contribute to nonadherence to topical treatment with TCSs [6,10]. Frequently, ‘natural’ remedies are labeled as amazingly efficacious for AD, creating further distrust of the standard therapy with TCSs [11]. Also, some alternative cures suggest that the causes of AD are minor issues that could be easily reversed, avoiding the implied triggers [11]. For instance, some creatively conceived pathogenetic theories of AD include an imaginative role of vaccines, diet, allergens, and chemicals in the etiopathogenesis of AD, contributing to erroneous beliefs and vague negative feelings about TCSs [11]. Most ironically, many corticophobic parents often apply apparently harmless Chinese herbal ointments with more ease than TCSs, hoping for positive effects, but actually placing themselves at increased risk of overdosing via the super-potent TCSs actually contained in these ointments [12].

The intense desire for effective treatment experienced by AD patients and their families renders them particularly susceptible to the overwhelming amount of misinformation about the efficacy and safety of TCSs. Also, they are susceptible to misinformation about the already mentioned alternative AD causes, therapies, and cures [11] that are frequently cited in the media. This can lead AD patients and their caregivers opting for nonconventional treatment options, to find quick fixes for their cutaneous issues that have great visual and psychosocial impact. Especially at risk of experiencing corticophobia are those who are more susceptible to pseudo-profound information, namely information that is encoded in profound sounding statements, which actually make very little sense [13]. Indeed, AD has a complex multifactorial aetiology, which is at higher risk of formulation of false myths and diffusion of misinformation, as many different systems are involved in the true disease aetiology, including skin-barrier dysfunction, T helper 2 cell-skewed immunity, and dysbiosis [11]. Notably, misinformation related to TCSs involves the fear of inducing a red skin syndrome, also known as ‘TCS withdrawal (TSW) syndrome’ or ‘TCS addiction syndrome’, with only few TCS applications. This leads many patients to try to manage their atopic eczema without using TCSs at all [9,14]. However, it must be specified that TSW syndrome is associated with the prolonged and regular application of TCSs, especially of high-strength TCSs [14]. TSW syndrome includes several different medical conditions, such as skin atrophy, rosacea, acne, and perioral dermatitis, possibly resulting from excessive TCS use over long periods of time. Also, problems experienced when stopping TCSs after using them for prolonged time are encompassed in the red skin syndrome, including rebound erythema and swelling, due to the enlargement of the previously vasoconstricted blood vessels [14]. Among adverse effects (AEs) from TCSs, skin atrophy must be mentioned, thus representing an uncommon localized AE, and it only occurs with repeated chronic application, usually for more than 12 months, at the same anatomical site, of a high-strength TCS. By all means, skin atrophy is reversible with cessation of TCS application [15,16]. Moreover, a recent review examining the safety of TCSs retrieved no evidence of skin thinning with the intermittent application of TCSs, used to treat acute flares of AD, or twice weekly, as ‘weekend therapy’, to prevent AD flares [17]. Regarding adrenal suppression or growth restriction, TCSs have negligible percutaneous absorption and are unlikely to cause systemic effects [8]. However, the application of a high-strength TCS to large areas of skin over a prolonged period can cause steroid absorption into the blood stream, and in rare cases this can lead to hypoadrenalism. Altogether, in a recent review of 16 clinical trials, assessing TCSs’ impact on the hypothalamic–pituitary–adrenal axis (HPAA), Levin et al. found no association between application of TCSs and HPAA suppression, concluding that TCSs are safe when used as recommended by current guidelines [18]. 

By all means, the incorrect or reduced use of TCSs deriving from corticophobia leads to lack of treatment response and to unsatisfactory therapeutic results, worsening the initial disbelief in TCSs and perpetuating the cycle of TCS demonization [19]. Assessing corticophobia and identifying risk factors for severe parental corticophobia are essential for comprehending the concerns and false beliefs that underlie this phenomenon, in order to address and allay these unfunded fears and ultimately augment treatment adherence.

## 2. Materials and Methods

Patients attending the Dermatology Unit of the pediatric hospital IRCCS Giannina Gaslini for skin examination were screened for AD. Patients with AD who visited from February 2023 to July 2023, and whose parents volunteered for completing the TOPICOP score questionnaire, were included in the study. All of them were pediatric (0–18 years). Patients were included in the study regardless of prior or actual steroid use and regardless of prior AD diagnosis.

Demographic data (age, sex, disease onset, previous healthcare professional consultations, parental educational degree, parental quality of life) were collected.

An EASI score was used to assess AD severity. An EASI score of ≤21 was considered mild to moderate, and a score of >21 was considered severe [20].

The Parental Dermatological Life Quality Index (DLQI) was assessed using the DLQI questionnaire, which was completed by one parent for each patient. Parental DLQI consists of a validated questionnaire designed for parents of patients with AD. A DLQI score of ≤10 was considered having a mild to moderate disease impact on parents’ life quality, and a score of ≥11 was considered severe [21].

Also, parents of patients with AD completed, on a voluntary basis, in autonomy, the self-administered questionnaire for the Topical Corticosteroid Phobia (TOPICOP) score, aimed at measuring corticophobia. The questionnaire was self-administered by parents, who completed the questionnaire alone, in absence of the dermatologist. The TOPICOP score is intended for use in daily practice and was recently translated into various languages, including Italian, and validated for international use [22]. It is based on 12 items, 6 concerning beliefs about TCSs and 6 concerning worries about TCSs, each scored on a 4-point Likert scale. The scale ranges from never to always (0  =  never, 1  =  sometimes, 2  =  often, 3  =  always) or from totally disagree to totally agree (0  =  totally disagree, 1  = do not really agree, 2  =  almost agree, 3  =  totally agree) depending on the question. For each parent, the global TOPICOP score was calculated as a percentage (0–100% TCS phobia) obtained from the sum of all responses divided by the maximum possible sum of included questions, multiplied by 100. The higher the TOPICOP score is, the more intense the corticophobia is. A TOPICOP score of ≤50% was considered mild to moderate, and a score of >50% was considered severe.

All subjects gave their informed consent for completing the TOPICOP score questionnaire for inclusion in the study. The study was conducted at IRCCS Gaslini retrospectively. The procedures followed were in accordance with the institutional ethical standards and the Helsinki Declaration of 1975, as revised in 1983.

### Statistical Analysis

A Fisher’s exact test, a χ^2^ test, and logistic regression were used to analyze data statistically. Statistical associations between parental corticophobia (mild/moderate vs. severe) and patients’ and parents’ characteristics were evaluated at first with the χ^2^ test or Fisher’s exact test when needed for small (<10) group size, to assess minor risk factors; *p* values < 0.05 were considered statistically significant. All variables were independent. Subsequently, logistic regression was used to identify major risk factors for corticophobia, among patients’ and parents’ characteristics; *p* values < 0.05 were considered statistically significant. All variables were independent.

The size of the sample was based on previous literature [5,6,23].

## 3. Results

### 3.1. Patients’ Features

Overall, 100 patients were included in the study (53 females; 47 males) with respectively one parent for each patient. Patients’ mean age was 5.9 years, with mean disease duration of 4.3 years (Table 1).

Regarding disease severity, a mean EASI score of 19.7 was registered. Of patients, 44% had mild to moderate disease (EASI ≤ 21), and 56% had severe (EASI > 21) disease.

Of the patients, 15% reported allergic rhinitis or rhinoconjunctivitis, and 4% presented allergic asthma.

Overall, 33% of patients had never consulted healthcare providers prior to the present visit, while 67% had already consulted healthcare professionals (Table 1).

Of the patients, 51% used no therapy at all for their eczema, 35% applied daily emollients only, 5% applied TCSs only, 4% applied daily emollients and TCSs during disease flares, and 5% used only oral steroids during diseases flare-ups.

### 3.2. Parents’ Features

DLQI questionnaires were completed by ninety-six mothers and four fathers of patients. DLQI scores of parents ranged from 0 to 30, with a mean value of 10.71 (95% CI from 9.53 to 11.89). Mothers scored on average 11, suggesting severe impact of the child’s disease on their life quality, while fathers scored on average 8, suggesting moderate impact of the child’s disease on their life quality.

Parental educational degree was low to intermediate in 60 cases and high (gymnasium or university degree) in 40 (Table 1).

#### Parental Corticophobia

The mean parental TOPICOP percentage score was 39.1%. Parental corticophobia was mild to moderate (TOPICOP ≤ 50%) for 51 patients, and it was severe (TOPICOP > 50%) for 49 (Table 1).

### 3.3. Associtation of Parental Corticophobia with Patients’ Characteristics

Severe parental corticophobia (TOPICOP > 50%) was registered in overall forty-nine parents of AD patients: twenty-five female patients and twenty-four male patients; seventeen very young (age ≤ 4 years) patients and thirty-two older (age >4 years) patients; six patients with severe AD (EASI >21) and forty-three patients with mild to moderate AD (EASI ≤ 21); three patients with late (after 1 year of age) disease onset and forty-six patients with early (prior to 1 year of age) disease onset; and eight patients with no previous healthcare professionals consultations and forty-one patients with previous healthcare professionals consultations.

Parental mild to moderate corticophobia (TOPICOP ≤ 50%) was registered in 51 parents of AD patients: 28 female patients and 23 male patients; 35 very young (age ≤ 4 years) patients and 16 older (age > 4 years) patients; 38 patients with severe AD (EASI >21) disease and 13 patients with mild to moderate AD (EASI ≤ 21); 20 patients with late (after 1 year of age) disease onset and 31 patients with early (prior to age 1 year) disease onset; and 25 patients with no previous healthcare professional consultations and 26 patients with previous healthcare professional consultations.

Spearman’s coefficient of rank correlation (rho) between the TOPICOP score (%) and EASI score stands at −0.539; significance level *p* < 0.0001; CI 95% for rho −0.665 to −0.383.

**Figure 1:**
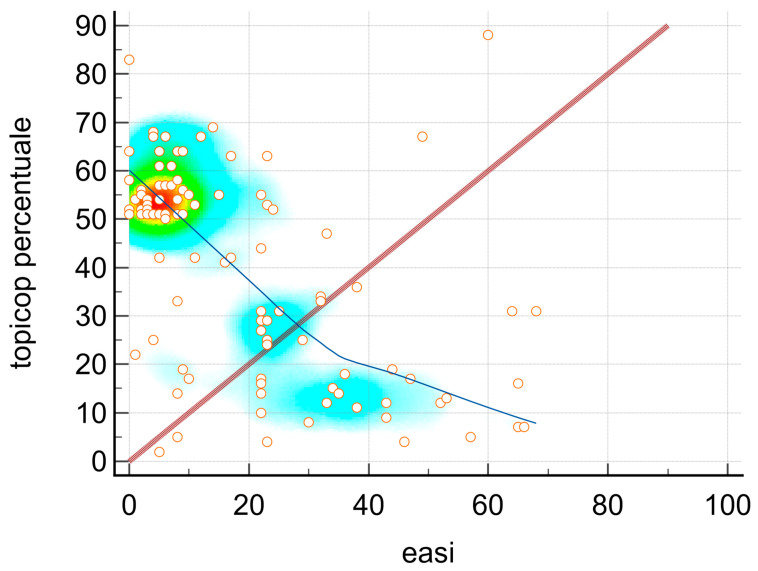
Spearman’s coefficient of rank correlation (rho) between TOPICOP score (%) and EASI score.

A proportionally inverse relationship between the TOPICOP score (%) and EASI score was evidenced (Figure 1). 

Severe parental corticophobia (TOPICOP > 50%) was very significantly associated with mild to moderate disease (43/49) (OR 20.9487; 95% CI 7.2489–60.5402; *p* < 0.001), while parents of children with severe AD had lower TOPICOP scores (38/51) (Table 2).

Also, severe parental corticophobia (TOPICOP > 50%) was very significantly associated with older age of patients (32/49) (OR 4.1176; 95% CI 1.7880 to 9.4828; *p* < 0.001), with early disease onset (46/49) (OR 9.8925; 95% CI 2.7064–36.1596; *p* < 0.001), and with previous healthcare professional consultations (41/49) (OR 4.9279; 95% CI 1.9335–12.5597; *p* < 0.001).

Severe parental corticophobia (TOPICOP > 50%) was not significantly associated with female or male sex (*p* = 0.1511) (Table 2).

### 3.4. Associtation of Parental Corticophobia with Parents’ Characteristics

Severe parental corticophobia (TOPICOP > 50%) was registered in forty parents with severe parental involvement of life quality (DLQI ≥ 11), nine parents with mild to moderate involvement of life quality (DLQI < 11), twenty-nine parents with high education (gymnasium or university degree), and twenty parents with low to intermediate education (Table 3).

Mild to moderate corticophobia (TOPICOP ≤ 50%) was registered in six parents with severe parental involvement of life quality (DLQI ≥ 11), forty-five parents with mild to moderate involvement of life quality (DLQI < 11), eleven parents with high educational degree (gymnasium or university degree), and forty parents with low to intermediate educational degree.

Severe parental corticophobia (TOPICOP > 50%) was very significantly associated with severe parental involvement of life quality (DLQI ≥ 11) (40/49) (OR 33.3333; 95% CI 10.9046–101.8937; *p* < 0.001), while mild to moderate corticophobia (TOPICOP ≤ 50%) was registered mainly in parents with mild to moderate involvement of life quality (DLQI < 11) (45/51) (Table 3).

Also, severe parental corticophobia (TOPICOP > 50%) was very significantly associated with high education of parents (gymnasium or university degree) (29/49) (OR 5.2727; 95% CI 2.1927–12.6790; *p* < 0.001), while mild to moderate corticophobia (TOPICOP ≤ 50%) was registered mainly in parents with low to intermediate educational degrees (40/51) (Table 3). 

### 3.5. Logistic Regression

With the logistic regression, all parameters already studied with the χ^2^ test and Fisher’s exact *t* test were analyzed. The logistic regression analysis examined the relationship between corticophobia and risk factors (independent variables) when all risk factors were considered together, permitting identification of the most influential risk factors for severe parental corticophobia [24]. Backward stepwise selection was used.

High parental DLQI (*p* < 0.0001), high parental education (*p* < 0.0338), older age of patients (*p* = 0.0015), and early disease onset (*p* < 0.0513) proved to be significantly associated with severe corticophobia (Table 4).

No statistical associations were found at logistic regression between severity of disease (EASI), gender of patients, and previous healthcare consultations and parental corticophobia (Table 4).

## 4. Discussion

In the present study, we found a number of risk factors that may be related to a higher TOPICOP score of corticophobia among parents of children with AD. The current findings may be subdivided into major risk factors, if identified using logistic regression analysis as most influential risk factors for corticophobia, and minor risk factors, at the χ^2^ test and Fisher’s exact *t* test only, for parental corticophobia. Interestingly, high parental education (OR 4.1; *p* < 0.0338) was identified as a major risk factor influencing corticophobia. This is in line with previous data from the literature, reporting widespread corticophobia among Italian parents with high educational degrees, leading to reduced treatment compliance and treatment failure. In their study, El Hachem et al. concluded there is a need to implement therapeutic education of families regarding the use of TCSs [23]. Notably, in contrast to El Hachem’s study, the present study retrieved mainly (60%) low educational degrees for parents of patients, while higher educational degrees (gymnasium or university degree) were recorded for only 40% of parents. Anyhow, the association between higher educational degree and higher corticophobia was confirmed (*p* < 0.001) by the present study, suggesting that parents with higher educational levels probably have more interest in or capacity for acquiring information about topical therapy for AD, yet the information, even if originating from healthcare professionals, may often be misleading. 

Also, data from the present study showed that a high parental DLQI is a statistically relevant major risk factor (OR 38.5; *p* < 0.0001) for severe parental corticophobia. This is not surprising, as a poor life quality of parents, measured by the parental dermatology life quality index, may also influence their judgement regarding therapies and their choices. 

Other relevant major risk factors for severe corticophobia were older age of patients (>4 years) (OR 14.5; *p* = 0.0015) and early disease onset (prior to 1 year of age) (OR 8.1; *p* < 0.0513), possibly because during the course of a long-lasting disease, contradictory information about TCSs may be gathered by the parents. Conversely, parents of young children (≤4 years) or with children presenting late disease onset (after 1 year of age) had on average lower phobias, probably because they had not yet built false preconceptions about the use of TCSs. 

As a confirmation of this, previous healthcare consultations were also accounted for as a risk factor in the present study, though minor, for severe parental corticophobia (*p* < 0.001). Undeniably, AD patients and their parents are informed about effects of TCSs in many different ways. TCS prescribers, including dermatologists, pediatricians, general practitioners, and pharmacists, should represent the most important source of information, but the internet, magazines, friends, and colleagues are also voices in the chorus. Moreover, while healthcare professionals should give truthful information about TCSs [1], they are not exempt from corticophobia, fueling the vicious circle of corticophobia. Indeed, in a recent study, Lambrecht et al. examined corticophobia among healthcare professionals using the TOPICOP questionnaire [7]. The study evidenced a high degree of corticophobia among healthcare professionals, especially among general practitioners and pharmacists, possibly influencing patients’ and parents’ perceptions of TCSs. Dermatologists showed the lowest levels of corticophobia, followed by pediatricians. Lambrecht et al. concluded that proper healthcare re-education is the first necessary step to increase general knowledge about TCSs and to improve the quality of information that healthcare professionals provide to patients and their parents, to ultimately improve the patients’ and parents’ compliance with TCS therapy [7]. Indeed, more education of general practitioners regarding TCS use, especially for children, is needed, as reported by Halewijn et al. [25] in a recent study conducted by interviewing 16 general practitioners [25]. What is more, Ragamin et al. reported an overall low adherence of general practitioners to recommendations and guidelines regarding TCS use in AD, as evidenced in a survey conducted among 391 general practitioners in the Netherlands [26].

Improvement of knowledge regarding TCSs could be possibly achieved on a large scale, as suggested by Ragamin et al. [27], through digital education of healthcare professionals. In their work, Ragamin et al. described the positive and encouraging effects of interactive online learning regarding healthcare professionals’ knowledge of TCSs [27]. We agree with Lambrecht and Ragamin and highlight the need to address corticophobia among healthcare professionals first and widely [7,27]. Indeed, data derived from the present study are in line with this concept, highlighting that parents with children subjected to previous consultations with healthcare professionals have the highest levels of corticophobia, (*p* < 0.001) possibly because of misleading information received from healthcare providers about TCSs. Of note, Koster et al. conducted an educational program about TCSs for pharmacy staff and parents and studied its effects, concluding that education and targeted counseling on the abovementioned categories are effective in reducing corticophobia [1]. Furthermore, Choi et al. recently evaluated the degree of corticophobia at their institute using the TOPICOP score and assessed patients’ levels of trust in sources from which they obtained information [3]. Interestingly, they observed that the most common source of information about TCSs was from dermatologists and that trust was highest in dermatologists. Also, they observed a median global TOPICOP score of 44.4%, higher for female than male patients, but with no significant differences based on severity of disease, age of patient, or educational level of parents [3].

In the present study, the severity of disease (EASI) accounted for a minor risk factor of severe parental corticophobia, and it influenced the TOPICOP score in inverse proportions. Altogether, parents of children with low to moderate disease severity (EASI ≤ 21) were among parental categories with the highest corticophobia (*p* < 0.001), suggesting that the subjective perception of disease accounts for most of the fears related to TCS therapy, rather than disease severity itself. As for parents of children with severe (EASI > 21) disease, they were more likely to use TCSs without fears, thirty-eight patients with severe AD (EASI > 21) had only low to moderate corticophobia, and only six patients with severe AD (EASI > 21) had severe corticophobia.

Study limitations comprise the absence of formal sample size calculations and the selection of patients based on the parents’ willingness to complete the TOPICOP score questionnaire. The sample size was adapted from other similar articles discussing corticophobia in parents of AD patients (e.g., Kojima et al., Lee et al., and El Hachem et al. recruited a sample size of similar order of magnitude as the present study) [5,6,23]. In particular, Lee et al. assessed corticophobia in 126 parents of children with AD in Korea (77.8% mothers, 22.2% fathers), similarly to our study, which evaluated a sample size of 100 (96% mothers, 4% fathers). 

## 5. Conclusions

In summary, we highlight the need to address parental corticophobia, assessing risk factors for severe corticophobia, in order to comprehend the origin of this complex phenomenon and especially to address groups of parents at higher risk for corticophobia by implementing educational programs. Parental categories more at risk for severe corticophobia are parents with high DLQI and high education, as well as parents of patients with long ongoing disease (older age of patients and early disease onset). These categories are more in need of correct education and information about TCSs. Providing clear information about TCSs, possibly supplementing verbal information with detailed written information about AD and TCSs, is essential to overcoming unfounded fears and ultimately augment treatment adherence and satisfaction and improve disease outcomes for their children with AD.

## Figures and Tables

**Table 1:** Features of patients and parents.

				Patients	Parents
N°				100	100 (one for every patient)
Sex (f = female; m = male)				53 f, 47 m	96 f, 4 m
Age (years)	mean			5.9 (95% CI from 5.2 to 6.7)	
	median			5.5 extreme values (3 months–16 years)	
Disease duration (years)	mean			4.3	
	median			3 extreme values (2 months–15 years)	
EASI	mean			19.7 (95% CI from 16.1 to 23.4)	
	median			13	
	EASI ≤ 21			44	
	EASI > 21			56	
Prior healthcare visits				67	
Previous therapy			No therapy	51	
			Daily emollients	35	
			TCS	5	
			daily emollients and TCS during disease flares	4	
			only oral steroids during diseases flares	5	
DLQI scores					10.71 (95% CI from 9.53 to 11.89). extreme values 0–30
Educational degree					60 low-intermediate; 40 high (gymnasium or university degree)
TOPICOP		mean			39.1% (95% CI from 34.8 to 43.4)
		median			48.5%
		TOPICOP ≤ 50%			51
		TOPICOP > 50%)			49

**Table 2:** Association of parental corticophobia with patients’ characteristics.

Patients	Parental Mild to Moderate Corticophobia (TOPICOP ≤ 50%)	Parental Severe Corticophobia (TOPICOP > 50%)	Significance Level	OR	95% CI
Total number of patients	51	49			
Female	28	25	*p* = 0.1511		
Male	23	24			
Age ≤ 4 years	35	17	*p* < 0.001	4.1176	1.7880–9.4828
Age > 4 years	16	32			
EASI > 21	38	6	*p* < 0.001	20.9487	7.2489–60.5402
EASI ≤ 21	13	43			
Onset after 1 year of age	20	3	*p* < 0.001	9.8925	2.7064–36.1596
Onset prior to 1 year of age	31	46			
No previous healthcare consultations	25	8	*p* < 0.001	4.9279	1.9335–12.5597
≥1 previous healthcare consultations	26	41			

**Table 3:** Association of parental corticophobia with parents’ characteristics.

Parents	Parental Mild to Moderate Corticophobia (TOPICOP ≤ 50%)	Parental Severe Corticophobia (TOPICOP >50%)	Significance Level	OR	95% CI
Total number of parents	51	49			
Parental DLQI ≥ 11	6	40	*p* < 0.001	33.3333	10.9046–101.8937
Parental DLQI < 11	45	9			
High education level (gymnasium or university degree),	11	29	*p* < 0.001	5.2727	2.1927–12.6790
Low/intermediate education level	40	20			

**Table 4:** Risk factors (independent variables) for corticophobia analyzed at logistic regression.

	*p*	Statistical Significance	OR	95% CI
Parental DLQI	<0.0001	*	38.5225	7.7225–192.1636
Parental education	<0.0338	*	4.1177	1.1144–15.2145
Age of patients	0.0015	*	14.5364	2.7818–75.9615
Disease onset	<0.0513	*	8.1052	0.9884–66.4655
EASI score	>0.05			
Gender of patients	>0.05			
Previous healthcare consultations	>0.05			

* = statistically significant.

## Data Availability

The data that support the findings of this study are available from the corresponding author (A.H.) upon reasonable request.
